# *μ*HEM for identification of differentially expressed miRNAs using hypercuboid equivalence partition matrix

**DOI:** 10.1186/1471-2105-14-266

**Published:** 2013-09-04

**Authors:** Sushmita Paul, Pradipta Maji

**Affiliations:** 1 Biomedical Imaging and Bioinformatics Lab, Indian Statistical Institute, 203, B. T. Road, Kolkata, 700108, India; 2 Machine Intelligence Unit, Indian Statistical Institute, 203, B. T. Road, Kolkata, 700108, India

**Keywords:** MicroRNA, Feature selection, Rough hypercuboid, Bootstrap error, Support vector machine

## Abstract

**Background:**

The miRNAs, a class of short approximately 22‐nucleotide non‐coding RNAs, often act post‐transcriptionally to inhibit mRNA expression. In effect, they control gene expression by targeting mRNA. They also help in carrying out normal functioning of a cell as they play an important role in various cellular processes. However, dysregulation of miRNAs is found to be a major cause of a disease. It has been demonstrated that miRNA expression is altered in many human cancers, suggesting that they may play an important role as disease biomarkers. Multiple reports have also noted the utility of miRNAs for the diagnosis of cancer. Among the large number of miRNAs present in a microarray data, a modest number might be sufficient to classify human cancers. Hence, the identification of differentially expressed miRNAs is an important problem particularly for the data sets with large number of miRNAs and small number of samples.

**Results:**

In this regard, a new miRNA selection algorithm, called *μ*HEM, is presented based on rough hypercuboid approach. It selects a set of miRNAs from a microarray data by maximizing both relevance and significance of the selected miRNAs. The degree of dependency of sample categories on miRNAs is defined, based on the concept of hypercuboid equivalence partition matrix, to measure both relevance and significance of miRNAs. The effectiveness of the new approach is demonstrated on six publicly available miRNA expression data sets using support vector machine. The.632+ bootstrap error estimate is used to minimize the variability and biasedness of the derived results.

**Conclusions:**

An important finding is that the *μ*HEM algorithm achieves lowest *B*.632+ error rate of support vector machine with a reduced set of differentially expressed miRNAs on four expression data sets compare to some existing machine learning and statistical methods, while for other two data sets, the error rate of the *μ*HEM algorithm is comparable with the existing techniques. The results on several microarray data sets demonstrate that the proposed method can bring a remarkable improvement on miRNA selection problem. The method is a potentially useful tool for exploration of miRNA expression data and identification of differentially expressed miRNAs worth further investigation.

## Background

The microRNAs or miRNAs are small non‐coding RNAs of length around 22 nucleotides, present in many plants and animals. They repress the expression of a gene post‐transcriptionally. In effect, they regulate expression of a gene or protein. The miRNAs are related to diverse cellular processes and regarded as important components of gene regulatory network. Studies into miRNA function have mainly focused on a variety of human diseases, particularly cancer, and mainly related to the use of miRNAs as disease biomarkers and for monitoring drug efficacy. Multiple reports have noted the utility of miRNAs for the diagnosis of cancer and other diseases [1].

Unlike with mRNAs, a modest number of miRNAs might be sufficient to classify human cancers [1]. Moreover, the bead‐based miRNA detection method has the attractive property of being not only accurate and specific, but also easy to implement in a routine clinical setting. In addition, unlike mRNAs, miRNAs remain largely intact in routinely collected, formalin‐fixed, paraffin‐embedded clinical tissues [2]. Recent studies have also shown that miRNAs can be detected in serum. These studies offer the promise of utilizing miRNA screening via less invasive blood‐based mechanisms. In addition, mature miRNAs are relatively stable. These phenomena make miRNAs superior molecular markers and targets for interrogation and as such, miRNA expression profiling can be utilized as a tool for cancer diagnosis and other diseases.

The functions of miRNAs appear to be different in various cellular functions. Just as miRNA is involved in the normal functioning of eukaryotic cells, so has dysregulation of miRNA been associated with disease [3]. It indicates that these miRNAs can prove to be potential biomarkers for developing a diagnostic tool. Hence, insilico identification of differentially expressed miRNAs that target genes involved in diseases is necessary. These differentially expressed miRNAs can be further used in developing effective diagnostic tools. Recently, few studies are carried out to identify differentially expressed miRNAs [4]‐[9]. However, absence of robust method makes it an open problem.

A miRNA expression data set can be represented by an expression table or matrix, where each row corresponds to one particular miRNA, each column to a sample, and each entry of the matrix is the measured expression level of a particular miRNA in a sample, respectively. However, for microarray data, the number of training samples is typically very small, while the number of miRNAs is in the thousands. Hence, the prediction rule formed by any classifier may not be able to be formed by using all available miRNAs. Even if all the miRNAs can be used, the use of all the miRNAs allows the noise associated with miRNAs of little or no discriminatory power, which inhibits and degrades the performance of the prediction rule in its application to unclassified or test samples. In other words, although the apparent error rate, which is the proportion of the training samples misclassified by the prediction rule, will decrease as it is formed from more and more miRNAs, its error rate in classifying samples outside of the training set eventually will increase. That is, the generalization error of the prediction rule will be increased if it is formed from a sufficiently large number of miRNAs. Hence, in practice, consideration has to be given to implement some procedure of feature selection for reducing the number of miRNAs to be used in constructing the prediction rule [10].

The method called significance analysis of microarrays is used in several works [11]‐[16] to identify differentially expressed miRNAs. Different statistical tests are also employed to identify differentially expressed miRNAs [1,4]‐[8,17]‐[20]. Xu et al. [21] used particle swarm optimization technique for selecting important miRNAs that contribute to the discrimination of different cancer types. However, one of the main problems in miRNA expression data analysis is uncertainty. Some of the sources of this uncertainty include imprecision in computations and vagueness in class definition. In this background, the rough set theory has gained popularity in modeling and propagating uncertainty. It deals with vagueness and incompleteness and is proposed for indiscernibility in classification according to some similarity [22]. It has been applied successfully to feature selection of discrete valued data [23]. Given a data set with discretized attribute values, it is possible to find a subset of the original attributes using rough set theory that are the most informative; all other attributes can be removed from the data set with minimal information loss. The theory of rough sets has also been successfully applied to microarray data analysis in [9,24]‐[35].

However, the real life high dimensional microarray data set may contain a number of irrelevant and insignificant miRNAs [9]. The presence of such miRNAs may lead to a reduction in useful information and degrade the prediction capability. The selected miRNA subset should contain the miRNAs those have high relevance with the classes and high significance in the miRNA set. Such miRNAs are expected to be able to predict the classes of the samples. Accordingly, a measure is required that can assess the effectiveness of a miRNA set [9].

In microarray data, the class labels of samples are represented by discrete symbols, while the expression values of miRNAs are continuous. Hence, to measure both relevance and significance of miRNAs using rough set theory, the continuous expression values of a miRNA have to be divided into several discrete partitions to generate equivalence classes [9]. However, the inherent error that exists in discretization process is of major concern in the computation of the dependency of real valued features. The rough hypercuboid approach of Wei et al. [36] is found to be suitable for numerical data sets.

In this regard, this paper presents a new miRNA selection method, termed as *μ*HEM. It employs rough hypercuboid approach to provide a means by which real valued noisy data can be effectively reduced without the need for user‐specified information. The proposed method selects a subset of miRNAs from whole miRNA set by maximizing both relevance and significance of the selected miRNAs. Using the concept of hypercuboid equivalence partition matrix, the degree of dependency is calculated for miRNAs, which is used to compute both relevance and significance of the miRNAs. Hence, the only information required in the proposed method is in the form of equivalence classes for each miRNA, which can be automatically derived from the data set. The concept of so‐called *B*.632+ error rate [37] is used to minimize the variability and biasedness of the derived results. The support vector machine is used to compute the *B*.632+ error rate as well as several other types of error rates as it maximizes the margin between data samples in different classes. The effectiveness of the proposed approach, along with a comparison with other related approaches, is demonstrated on several miRNA expression data sets.

## Methods

### Data sets used

In the current research work, publicly available six miRNA expression data sets with accession number GSE17681, GSE17846, GSE21036, GSE24709, GSE28700, and GSE31408 are used, which are downloaded from Gene Expression Omnibus (http://www.ncbi.nlm.nih.gov/geo/).

#### GSE17681

This data set has been generated to detect specific patterns of miRNAs in peripheral blood samples of lung cancer patients. As controls, blood of donors without known affection have been tested. The number of miRNAs, samples, and classes in this data sets are 866, 36, and 2, respectively [38].

#### GSE17846

This data set represents the analysis of miRNA profiling in peripheral blood samples of multiple sclerosis and in the blood of normal donors. It contains 864 miRNAs, 41 samples, and 2 classes [39].

#### GSE21036

This data set contains miRNA expression profiles of 218 prostate tumors with primary or metastatic prostate cancer with a median of 5 years clinical follow‐up. The number of miRNAs and samples are 373 and 141, respectively [40].

#### GSE24709

It analyzes peripheral miRNA blood profiles of patients with lung diseases. The miRNA expression profiling has been done for patients with lung cancer, chronic obstructive pulmonary disease, and normal controls. It contains total 863 miRNAs, 71 samples, and 3 classes.

#### GSE28700

This data set contains expression profiles of miRNAs from 22 paired gastric cancer and normal tissues. It contains total 44 samples and 470 miRNAs. The samples are grouped into 2 classes [41].

#### GSE31408

It analyzes miRNA expression profiles of cutaneous T‐cell lymphomas and benign inflammation of skin. It consists of total 705 miRNAs, 148 samples, and 2 classes [42].

### Method

#### Hypercuboid equivalence partition matrix

Let $U=\{x_{1},\cdots \;,x_{i},\cdots \;,x_{n}\}$ be the set of *n* objects or samples and $C=\{A_{1},\cdots \;,A_{i},\cdots \;,A_{j},\cdots \;,A_{m}\}$ denotes the set of *m* attributes or miRNAs of a given microarray data set $T=\{w_{\text{ij}}|i=1,\cdots \;,m,j=1,\cdots \;,n\}$, where *w*_*ij*_∈ℜ is the measured expression value of the miRNA $A_{i}$ in the sample *x*_*j*_. Let D be the set of class labels or sample categories of *n* samples. In rough set theory, the attribute sets C and D are termed as the condition and decision attribute sets in U, respectively.

If $U/D=\{\beta_{1},\cdots \;,\beta_{i},\cdots \;,\beta_{c}\}$ denotes *c* equivalence classes or information granules of U generated by the equivalence relation induced from the decision attribute set D, then *c* equivalence classes of U can also be generated by the equivalence relation induced from each condition attribute $A_{k}\in C$. If $U/A_{k}=\{\delta_{1},\cdots \;,\delta_{i},\cdots \;,\delta_{c}\}$ denotes *c* equivalence classes or information granules of U induced by the condition attribute $A_{k}$ and *n* is the number of objects in U, then *c*‐partitions of U are the sets of (*cn*) values $\left\{h_{\text{ij}}(A_{k})\right\}$ that can be conveniently arrayed as a (*c*×*n*) matrix $H(A_{k})=[h_{\text{ij}}(A_{k})]$. The matrix $H(A_{k})$ is denoted by

(1) $$H(A_{k})=\left(\begin{array}{llll} h_{11}(A_{k}) & h_{12}(A_{k}) & \cdots & h_{1n}(A_{k})\\ h_{21}(A_{k}) & h_{22}(A_{k}) & \cdots & h_{2n}(A_{k})\\ \cdots & \cdots & \cdots & \cdots \\ h_{c1}(A_{k}) & h_{c2}(A_{k}) & \cdots & h_{\text{cn}}(A_{k}) \end{array}\right)$$

(2) $$\text{where}\;\;h_{\text{ij}}(A_{k})=\left\{\begin{array}{ll} 1 & \text{if}L_{i}\leq x_{j}(A_{k})\leq U_{i}\\ 0 & \text{otherwise.} \end{array}\right)$$

The tuple [L_*i*_,U_*i*_] represents the interval of *i*th class *β*_*i*_ according to the decision attribute set D. The interval [L_*i*_,U_*i*_] is the value range of condition attribute $A_{k}$ with respect to class *β*_*i*_. It is spanned by the objects with same class label *β*_*i*_. That is, the value of each object *x*_*j*_ with class label *β*_*i*_ falls within interval [L_*i*_,U_*i*_]. This can be viewed as a supervised granulation process, which utilizes class information.

Generally, an *m*‐dimensional hypercuboid or hyperrectangle is defined in the *m*‐dimensional Euclidean space, where the space is defined by the *m* variables measured for each sample or object. In geometry, a hypercuboid or hyperrectangle is the generalization of a rectangle for higher dimensions, formally defined as the Cartesian product of orthogonal intervals. A *d*‐dimensional hypercuboid with *d* attributes as its dimensions is defined as the Cartesian product of *d* orthogonal intervals. It encloses a region in the *d*‐dimensional space, where each dimension corresponds to a certain attribute. The value domain of each dimension is the value range or interval that corresponds to a particular class.

The *c*×*n* matrix $H(A_{k})$ is termed as hypercuboid equivalence partition matrix of the condition attribute $A_{k}$. It represents the *c*‐hypercuboid equivalence partitions of the universe generated by an equivalence relation. Each row of the matrix $H(A_{k})$ is a hypercuboid equivalence partition or class. Here $h_{\text{ij}}(A_{k})\in \{0,1\}$ represents the membership of object *x*_*j*_ in the *i*th equivalence partition or class *β*_*i*_ satisfying following two conditions:

(3) $$\begin{array}{ll} & 1\leq \overset{n}{\underset{j=1}{\sum }}h_{\text{ij}}(A_{k})\leq n,\forall i;\; \end{array}$$

(4) $$\begin{array}{ll} & 1\leq \overset{c}{\underset{i=1}{\sum }}h_{\text{ij}}(A_{k})\leq c,\forall \mathrm{j.}\; \end{array}$$

The above axioms should hold for every equivalence partition, which correspond to the requirement that an equivalence class is non‐empty. However, in real data analysis, uncertainty arises due to overlapping class boundaries. Hence, such a granulation process does not necessarily result in a compatible granulation in the sense that every two class hypercuboids or intervals may intersect with each other. The intersection of two hypercuboids also forms a hypercuboid, which is referred to as implicit hypercuboid. The implicit hypercuboids encompass the misclassified samples or objects those belong to more than one classes. The degree of dependency of the decision attribute set or class label on the condition attribute set depends on the cardinality of the implicit hypercuboids. The degree of dependency increases with the decrease in cardinality. Hence, the degree of dependency of decision attribute on a condition attribute set is evaluated by finding the implicit hypercuboids that encompass misclassified objects. Using the concept of hypercuboid equivalence partition matrix, the misclassified objects of implicit hypercuboids can be identified based on the confusion vector defined next

(5) $$\begin{array}{ll} & V(A_{k})=\;[\;v_{1}(A_{k}),\cdots \;,v_{j}(A_{k}),\cdots \;,v_{n}(A_{k})]\; \end{array}$$

(6) $$\begin{array}{ll} & \text{where}\;v_{j}(A_{k})=\min\{1,\overset{c}{\underset{i=1}{\sum }}h_{\text{ij}}(A_{k})-1\}.\; \end{array}$$

According to the rough set theory, if an object *x*_*j*_ belongs to the lower approximation of any class *β*_*i*_, then it does not belong to the lower or upper approximations of any other classes and $v_{j}(A_{k})=0$. On the other hand, if the object *x*_*j*_ belongs to the boundary region of more than one classes, then it should be encompassed by the implicit hypercuboid and $v_{j}(A_{k})=1$. Hence, the hypercuboid equivalence partition matrix and corresponding confusion vector of the condition attribute $A_{k}$ can be used to define the lower and upper approximations of the *i*th class *β*_*i*_ of the decision attribute set D.

Let $\beta_{i}\subseteq U$. *β*_*i*_ can be approximated using only the information contained within $A_{k}$ by constructing the *A*‐lower and *A*‐upper approximations of *β*_*i*_:

(7) $$\underline{A}(\beta_{i})=\{x_{j}|\;h_{\text{ij}}(A_{k})=1\;\text{and}\;v_{j}(A_{k})=0\};$$

(8) $$\begin{array}{c} \bar{A}(\beta_{i})=\{x_{j}|\;h_{\text{ij}}(A_{k})=1\}; \end{array}$$

where equivalence relation *A* is induced from attribute $A_{k}$. The boundary region of *β*_*i*_ is then defined as

(9) $$\begin{array}{c} {\text{BN}}_{A}(\beta_{i})=\{x_{j}|\;h_{\text{ij}}(A_{k})=1\;\text{and}\;v_{j}(A_{k})=1\}. \end{array}$$

#### Dependency

Combining (1), (5), and (7), the dependency between condition attribute $A_{k}$ and decision attribute D can be defined as follows:

(10) $$\begin{array}{ll} & \gamma_{A_{k}}(D)=\frac{1}{n}\overset{c}{\underset{i=1}{\sum }}\overset{n}{\underset{j=1}{\sum }}h_{\text{ij}}(A_{k})\;\cap \;[1-v_{j}(A_{k})],\; \end{array}$$

(11) $$\begin{array}{ll} & \text{that is,}\;\gamma_{A_{k}}(D)=1-\frac{1}{n}\overset{n}{\underset{j=1}{\sum }}v_{j}(A_{k}),\;\;\;\;\;\; \end{array}$$

where $0\leq \gamma_{A_{k}}(D)\leq 1$. If $\gamma_{A_{k}}(D)=1$, D depends totally on $A_{k}$, if $0<\gamma_{A_{k}}(D)<1$, D depends partially on $A_{k}$, and if $\gamma_{A_{k}}(D)=0$, then D does not depend on $A_{k}$. The $\gamma_{A_{k}}(D)$ is also termed as the relevance of attribute $A_{k}$ with respect to class D.

#### Significance

Given two condition attributes $A_{k}$ and $A_{l}$, the *c*×*n* hypercuboid equivalence partition matrix corresponding to the set $A=\{A_{k},A_{l}\}$ can be calculated from two *c*×*n* hypercuboid equivalence partition matrices $H(A_{k})$ and $H(A_{l})$ as follows:

(12) $$\begin{array}{ll} & H(\{A_{k},A_{l}\})=H(A_{k})\cap H(A_{l});\;\;\;\;\;\;\;\; \end{array}$$

(13) $$\begin{array}{ll} & \text{where}\;h_{\text{ij}}(\{A_{k},A_{l}\})=h_{\text{ij}}(A_{k})\cap h_{\text{ij}}(A_{l}).\; \end{array}$$

The change in dependency when an attribute is removed from the set of condition attributes, is a measure of the significance of the attribute. To what extent an attribute is contributing to calculate the dependency on decision attribute can be calculated by the significance of that attribute. The significance of the attribute $A_{k}$ with respect to the condition attribute set $\left\{A_{k},A_{l}\right\}$ is given by

(14) $$\begin{array}{c} \sigma_{A}(D,A_{k})=\frac{1}{n}\overset{n}{\underset{j=1}{\sum }}\left[v_{j}(A-\{A_{k}\})-v_{j}(A)\right]; \end{array}$$

where $0\leq \sigma_{\left\{A_{k},A_{l}\right\}}(D,A_{k})\leq 1$. Hence, the higher the change in dependency, the more significant the attribute $A_{k}$ is. If significance is 0, then the attribute is dispensable.

#### ***μ***HEM: proposed miRNA selection method

Let $\gamma_{A_{i}}(D)$ be the relevance of the miRNA $A_{i}$ with respect to the class labels D and $\sigma_{\left\{A_{i},A_{j}\right\}}(D,A_{i})$ is the significance of the miRNA $A_{i}$ with respect to another miRNA $A_{j}\in S$, where S is the set of selected miRNAs. The average relevance of all selected miRNAs is, therefore, given by

(15) $$\begin{array}{c} J_{\text{relev}}=\frac{1}{\left|S\right|}\underset{A_{i}\in S}{\sum }\gamma_{A_{i}}(D), \end{array}$$

while the average significance among the selected miRNAs is as follows

(16) $$\begin{array}{ll} J_{\text{signf}}= & \frac{1}{\left|S|(|S|-1\right)}\\ & \times \underset{A_{i}\neq A_{j}\in S}{\sum }\{\sigma_{\left\{A_{i},A_{j}\right\}}(D,A_{i})+\sigma_{\left\{A_{i},A_{j}\right\}}(D,A_{j})\}. \end{array}$$

Therefore, the problem of selecting a set S of relevant and significant miRNAs from the whole miRNA set C is equivalent to maximize $J_{\text{relev}}$ and $J_{\text{signf}}$, that is, to maximize the objective function J, where

(17) $$J=\omega J_{\text{relev}}+(1-\omega )J_{\text{signf}}$$

where *ω* is a weight parameter. To solve the above problem, the following greedy algorithm is used. 

1. Initialize $C\leftarrow \{A_{1},\cdots \;,A_{i},\cdots \;,A_{m}\},S\leftarrow \emptyset$.

2. Generate hypercuboid equivalence partition matrix $H(A_{i})$ and corresponding confusion vector $V(A_{i})$ for each miRNA $A_{i}\in C$ using (1) and (5), respectively.

3. Calculate the relevance $\gamma_{A_{i}}(D)$ of each miRNA $A_{i}\in C$ using (11).

4. Select the miRNA $A_{i}$ as the most relevant miRNA that has highest relevance value $\gamma_{A_{i}}(D)$. In effect, $A_{i}\in S$ and $C=C\setminus A_{i}$.

5. Repeat the following two steps until C=∅ or the desired number of miRNAs is selected.

6. Repeat the following four steps for each of the remaining miRNAs of C. 

(a) Generate hypercuboid equivalence partition matrix $H(\{A_{i},A_{j}\})$ using (12) between each selected miRNA $A_{i}\in S$ and each miRNA $A_{j}\in C$.

(a) Generate corresponding confusion vector $V(\{A_{i},A_{j}\})$ for two miRNAs $A_{i}$ and $A_{j}$ using (5).

(a) Calculate the significance of each miRNA $A_{j}\in C$ with respect to each of the already selected miRNAs of S using (14).

(a) Remove $A_{j}$ from C if it has zero significance value with respect to any one of the selected miRNAs. In effect, $C=C\setminus A_{j}$.

7. From the remaining miRNAs of C, select miRNA $A_{j}$ that maximizes the following condition:

(18) $$\omega \gamma_{A_{j}}(D)+\frac{\left(1-\omega \right)}{\left|S\right|}\underset{A_{i}\in S}{\sum }\sigma_{\left\{A_{i},A_{j}\right\}}(D,A_{j}).$$

As a result of that, $A_{j}\in S$ and $C=C\setminus A_{j}$.

8. Stop.

#### Computational complexity

The proposed *μ*HEM method has low computational complexity with respect to the number of miRNAs, samples, and classes. Prior to computing the relevance or significance of a miRNA, the hypercuboid equivalence partition matrix and confusion vector for each miRNA are to be generated first, which are carried out in Step 2 of the proposed algorithm. The computational complexity to generate a (*c*×*n*) hypercuboid equivalence partition matrix is O(cn), where *c* and *n* represent the number of classes and objects in the data set, respectively, while the generation of confusion vector has also O(cn) time complexity. In effect, the computation of the relevance of a miRNA has O(cn) time complexity. Hence, the total complexity to compute the relevance of *m* miRNAs, which is carried out in Step 3 of the proposed algorithm, is O(mcn). The selection of most relevant miRNA from the set of *m* miRNAs, which is carried out in Step 4, has a complexity O(m).

There is only one loop in Step 5 of the proposed miRNA selection method, which is executed (*d*−1) times, where *d* represents the number of selected miRNAs. The complexity to compute the significance of a candidate miRNA with respect to another miRNA has also the complexity O(cn). If $\overset{´}{m}$ represents the cardinality of the already selected miRNA set, the total complexity to compute the significance of $\left(m-\overset{´}{m}\right)$ candidate miRNAs, which is carried out in Step 6, is $O((m-\overset{´}{m})\text{cn})$. The selection of a miRNA from $\left(m-\overset{´}{m}\right)$ candidate miRNAs by maximizing relevance and significance, which is carried out in Step 7, has a complexity $O(m-\overset{´}{m})$. Hence, the total complexity to execute the loop (*d*−1) times is $\left(O((d-1)((m-\overset{´}{m})+(m-\overset{´}{m})\text{cn}))=)O(\text{dcn}(m-\overset{´}{m})\right)$.

In effect, the selection of a set of *d* relevant and significant miRNAs from the whole set of *m* miRNAs using the proposed hypercuboid equivalence partition matrix based first order incremental search method has an overall computational complexity of $\left(O(\text{mcn})+O(m)+O(\text{dcn}(m-\overset{´}{m}))=)O(\text{dnm}\right)$ as $c,\overset{´}{m}<<m$.

#### B.632+ error rate

In order to minimize the variability and biasedness of derived result, the so‐called *B*.632+ bootstrap approach [37] is used, which is defined as follows:

(19) $$\mathrm{B.}632+=(1-\tilde{\omega })\text{AE}+\tilde{\omega }B1$$

where *AE* denotes the proportion of the original training samples misclassified, termed as apparent error rate, and *B*1 is the bootstrap error, defined as follows:

(20) $$B1=\frac{1}{n}\overset{n}{\underset{j=1}{\sum }}\left(\frac{\overset{M}{\underset{k=1}{\sum }}I_{\text{jk}}Q_{\text{jk}}}{\overset{M}{\underset{k=1}{\sum }}I_{\text{jk}}}\right)$$

where *n* is the number of original samples and M is the number of bootstrap samples. If the sample *x*_*j*_ is not contained in the *k*th bootstrap sample, then *I*_*jk*_=1, otherwise 0. Similarly, if *x*_*j*_ is misclassified, *Q*_*jk*_=1, otherwise 0. The weight parameter $\tilde{\omega }$ is given by

(21) $$\tilde{\omega }=\frac{0.632}{1-0.368r};$$

(22) $$\text{where}\;r=\frac{B1-\text{AE}}{\gamma -\text{AE}};$$

(23) $$\text{and}\;\gamma =\overset{c}{\underset{i=1}{\sum }}p_{i}(1-q_{i});$$

where *c* is the number of classes, *p*_*i*_ is the proportion of the samples from the *i*th class, and *q*_*i*_ is the proportion of them assigned to the *i*th class. Also, *γ* is termed as the no‐information error rate that would apply if the distribution of the class‐membership label of the sample *x*_*j*_ did not depend on its feature vector.

#### Support vector machine

In the current study, the support vector machine (SVM) [43] is used to evaluate the performance of the proposed *μ*HEM algorithm as well as several other feature selection algorithms. The SVM is a margin classifier that draws an optimal hyperplane in the feature vector space; this defines a boundary that maximizes the margin between data samples in different classes, therefore leading to good generalization properties. A key factor in the SVM is to use kernels to construct nonlinear decision boundary. In the present work, linear kernels are used. The source code of the SVM has been downloaded from Library for Support Vector Machines (http://www.csie.ntu.edu.tw/~cjlin/libsvm/).

To compute different types of error rates obtained using the SVM, bootstrap approach is performed on each miRNA expression data set. For each training set, a set of differential miRNAs is first generated, and then the SVM is trained with the selected miRNAs. After the training, the information of miRNAs those were selected for the training set is used to generate test set and then the class label of the test sample is predicted using the SVM. For each data set, fifty top‐ranked miRNAs are selected for the analysis.

In order to calculate the *B*.632+ error rate, apparent error (*AE*) is first calculated. This error is obtained when the same original data set is used to train and test a classifier. After that, the *B*1 error is computed from M bootstrap samples. Finally, the no‐information error (*γ*) is calculated by randomly perturbing the class label of a given data set. The mutated data set is used for miRNA selection and the selected miRNA set is used to build the SVM. Then, the trained SVM is used to classify the original data set. The error generated by this procedure is known as *γ* rate. Finally, the *B*.632+ error rate is computed based on the *AE*, *B*1 error, and *γ* error using (19).

## Results and discussions

The performance of the proposed hypercuboid equivalence partition matrix based miRNA selection (*μ*HEM) method is extensively studied and compared with that of some existing feature selection algorithms. The algorithms compared are mutual information based InfoGain [44] and minimum redundancy‐maximum relevance (mRMR) algorithm [45], method proposed by Golub et al. [46], rough set based maximum relevance‐maximum significance (RSMRMS) algorithm [9,28], boosting [47] and lasso [48]. The source code of the proposed *μ*HEM algorithm, written in C language, is available at http://www.isical.ac.in/~bibl/results/mihem/mihem.html. All the algorithms are run in Ubuntu 12.04 LTS having machine configuration Intel Core i7‐2600 CPU @ 3.40GHz × 8, and 16 GB RAM.

### Performance analysis of ***μ***HEM algorithm

This section presents the performance of the proposed *μ*HEM algorithm on six miRNA data sets with respect to the *B*.632+ error rate of the SVM.

#### Optimum value of weight parameter ***ω***

The weight parameter *ω* in (18) regulates the relative importance of the significance of the candidate miRNA with respect to the already selected miRNAs and the relevance with the output class. If *ω* is one, only the relevance with the output class is considered for each miRNA selection. The presence of a *ω* value lower than one is crucial in order to obtain good results. If the significance between miRNAs is not taken into account, selecting the miRNAs with the highest relevance with respect to the output class may tend to produce a set of redundant and insignificant miRNAs that may leave out useful complementary information. On the other hand, if *ω* is zero, the miRNAs are selected based on their significance values only without considering the relevance of each miRNA. In effect, the selected miRNA set may contain a number of irrelevant miRNAs. Hence, the value of weight parameter *ω* should be in between zero and one in order to obtain good results, that is, 0<*ω*<1.

To find out the optimum value of *ω* for each miRNA data set, the coefficient of variation (*C*_*v*_) of average significance value is used. It is a measure of relative dispersion and defined as a quotient between standard deviation and mean value. Let the average significance value of the *j*th selected miRNA $A_{j}$ with respect to the already selected miRNA set $S_{j-1}$, for a given *ω* value, be

(24) $$\Omega_{j}(\omega )=\frac{1}{\left|S_{j-1}\right|}\underset{\begin{array}{c} A_{i}\neq A_{j}\in S_{j-1}\\ j>i \end{array}}{\sum }\sigma_{\left\{A_{i},A_{j}\right\}}(D,A_{j})$$

where D represents the set of class labels of the samples and $S_{j}=S_{j-1}\cup \{A_{j}\}$. If *μ*(*ω*) and *s*(*ω*) represent the mean and standard deviation of the average significance values of *d* selected miRNAs for a particular value of *ω*, then the *C*_*v*_ index is defined as follows:

(25) $$C_{v}(\omega )=\frac{s(\omega )}{\mu (\omega )};$$

where mean and standard deviation for *d* selected miRNAs are computed as follows:

(26) $$\mu (\omega )=\frac{1}{d}\overset{d}{\underset{i=1}{\sum }}\Omega_{i}(\omega );$$

(27) $$s(\omega )=\sqrt{\frac{1}{d}\overset{d}{\underset{i=1}{\sum }}{\left[\mu (\omega )-\Omega_{i}(\omega )\right]}^{2}}.$$

The lower value of the *C*_*v*_ index, that is, the higher value of mean *μ* and lower value of standard deviation *s*, ensures that the average significance of the set of selected miRNAs is higher. A good miRNA selection method should make the value of *C*_*v*_ index as low as possible.

**Figure 1:**
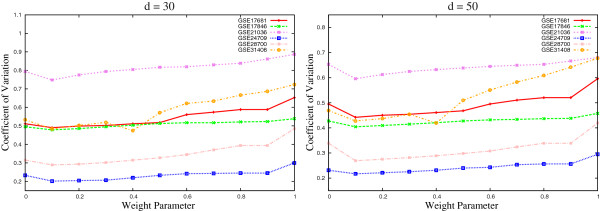
**Variation of the *C***_***v***_** index for different values of weight parameter *ω*.**

To find out the optimum value of *ω*, extensive experimentation is carried out on six miRNA expression data sets. The value of *ω* is varied from 0.0 to 1.0. In the current study, *d*=30 and *d*=50 top‐ranked miRNAs are selected for analysis. Figure 1 presents the variation of the *C*_*v*_ index obtained using the proposed *μ*HEM algorithm for different values of *ω* on six miRNA data sets. From the results reported in Figure 1, it is seen that as the value of weight parameter *ω* increases, the *C*_*v*_ index decreases and attains its minimum value at a particular value of *ω*=*ω*^⋆^. After that the *C*_*v*_ index value increases with the increase in the value of *ω*. Hence, the optimum value of *ω* for each data set is obtained using the following relation:

(28) $$\omega^{⋆}=\arg\;\underset{\omega }{\min}\left\{C_{v}(\omega )\right\}.$$

The optimum values of *ω* obtained using (28) are 0.1 for GSE17681, GSE17846, GSE21036, GSE24709, and GSE28700, and 0.4 for GSE31408, irrespective of the number of selected miRNAs.

**Figure 2:**
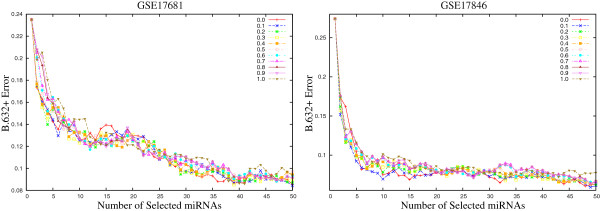
**Variation of *B.632+ *error rate on GSE17681 and GSE17846 data sets for different values of weight parameter *ω∈ [ 0.0,1.0] *averaged over 50 random splits.**

**Figure 3:**
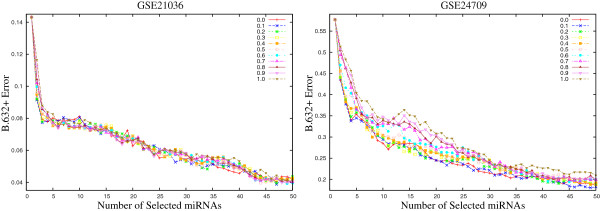
**Variation of *B.632+ *error rate on GSE21036 and GSE24709 data sets for different values of weight parameter *ω∈ [ 0.0,1.0] *averaged over 50 random splits.**

Figures 2 and 3 present the variation of the *B*.632+ error rate obtained using the proposed *μ*HEM algorithm for different values of *ω* on GSE17681, GSE17846, GSE21036, and GSE24709 data sets as examples considering *d*=50. From the results reported in Figures 2 and 3, it is seen that the *B*.632+ error rate of the SVM decreases with the increase in the number of selected miRNAs, irrespective of the value of *ω*. Also, the error rate is lower for 0.0<*ω*<0.5 than both *ω*=0.0 and 1.0. Similar results can also be seen for both GSE28700 and GSE31408 data sets.

Finally, Table 1 presents the minimal *B*.632+ error rate of the SVM for different values of weight parameter *ω*, along with the value of *C*_*v*_ index. For each miRNA data set, the minimum *B*.632+ error rate is written in italic, while the best *C*_*v*_ index is marked in bold. From the results reported in Table 1, it is seen that the proposed *μ*HEM algorithm achieves its best performance at *ω*=*ω*^⋆^ in five cases out of total six miRNA data sets. Only for GSE28700 data set, the *B*.632+ error rate at *ω*=*ω*^⋆^ is higher than that of both *ω*=0.0 and 1.0. The lowest *B*.632+ error rate is achieved at *ω*=1.0 for this data set. All the results reported in Figures 1, 2, and 3, and Table 1 establish the importance of both relevance and significance criteria in the proposed *μ*HEM method for selecting differentially expressed miRNAs from a microarray data.

**Table 1:** **Performance of *μ*HEM algorithm on six miRNA data sets for different values of *ω***

**Value**	**GSE17681**		**GSE17846**		**GSE21036**		**GSE24709**		**GSE28700**		**GSE31408**	
**of *ω***	***B.632+***	***C***_***v***_	***B.632+***	***C***_***v***_	***B.632+***	***C***_***v***_	***B.632+***	***C***_***v***_	***B.632+***	***C***_***v***_	***B.632+***	***C***_***v***_
0.0	0.0854	0.4951	0.0605	0.4275	0.0403	0.6528	0.1863	0.2312	0.2498	0.3388	0.0757	0.4688
0.1	*0.0842*	**0.4421**	*0.0590*	**0.4042**	*0.0388*	**0.5956**	*0.1803*	**0.2171**	0.2566	**0.2693**	0.0753	0.4275
0.2	0.0870	0.4502	0.0623	0.4094	0.0396	0.6124	0.1898	0.2213	0.2660	0.2752	0.0742	0.4368
0.3	0.0851	0.4542	0.0644	0.4148	0.0410	0.6246	0.1878	0.2256	0.2572	0.2818	0.0732	0.4543
0.4	0.0894	0.4611	0.0627	0.4206	0.0420	0.6319	0.1881	0.2312	0.2583	0.2889	*0.0672*	**0.4190**
0.5	0.0882	0.4680	0.0640	0.4275	0.0394	0.6384	0.1970	0.2399	0.2587	0.2980	0.0690	0.5097
0.6	0.0882	0.4951	0.0651	0.4319	0.0392	0.6447	0.1940	0.2429	0.2571	0.3079	0.0693	0.5508
0.7	0.0893	0.5105	0.0637	0.4337	0.0402	0.6493	0.1951	0.2536	0.2632	0.3241	0.0683	0.5826
0.8	0.0893	0.5202	0.0636	0.4366	0.0405	0.6528	0.1992	0.2564	0.2649	0.3388	0.0690	0.6088
0.9	0.0893	0.5202	0.0636	0.4380	0.0398	0.6664	0.2002	0.2564	0.2650	0.3388	0.0697	0.6414
1.0	0.0860	0.5958	0.0724	0.4575	0.0410	0.6801	0.2095	0.2950	*0.2475*	0.4191	0.0693	0.6771

#### Optimum number of selected miRNAs

According to Lu et al. [1], unlike with mRNAs, a modest number of miRNAs might be sufficient to classify human cancers. Also, the number of training samples is typically very small compare to the number of miRNAs. Hence, the use of large number of miRNAs in constructing classifier may degrade the prediction capability on test samples [10].

**Figure 4:**
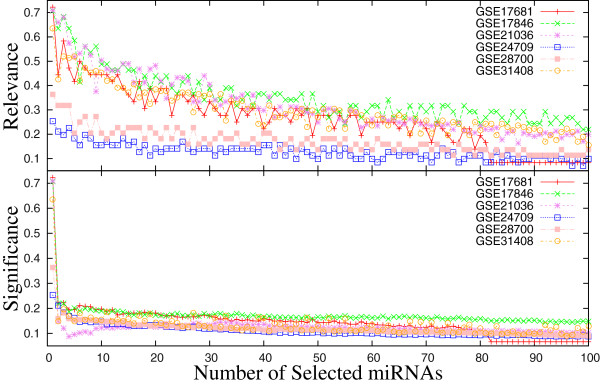
Relevance and significance values of each of the selected miRNAs for different miRNA data sets.

In order to find out the optimum number of selected miRNAs, extensive experimentation is carried out on six microarray data sets. Figure 4 depicts the relevance and average significance values of each of the selected miRNAs for six expression data sets. The results are presented for optimum values of *ω* considering 100 selected miRNAs. From the results reported in Figure 4, it can be seen that as the number of selected miRNAs increases, both relevance and significance values decrease. Also, the significance value remains constant after selecting forty to forty‐five miRNAs, irrespective of the data sets used. Hence, in the current study, the selected number of miRNAs is set to *d*=50.

#### Error rate and execution time

**Figure 5:**
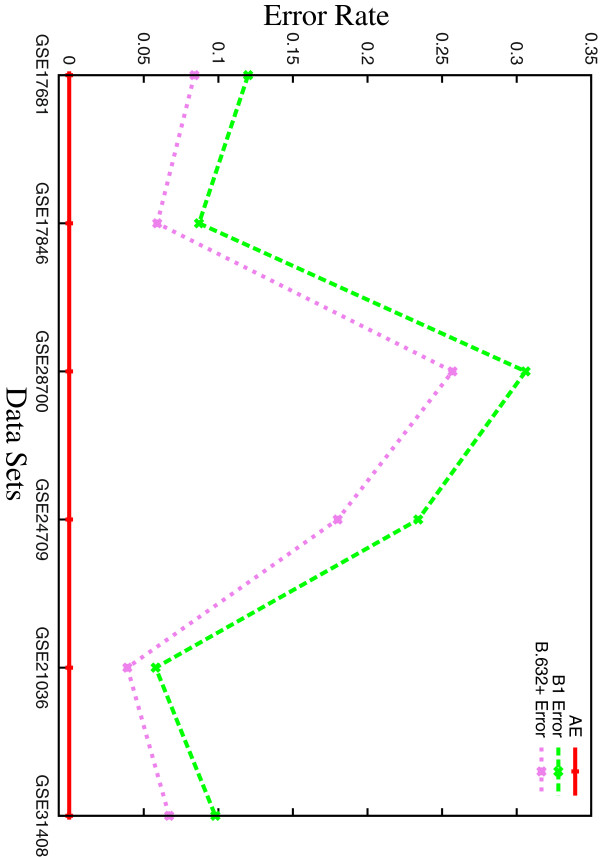
**Variation of several error rates obtained using *μ*HEM algorithm for different number of samples.**

Figure 5 presents the variation of several error rates obtained using the proposed *μ*HEM algorithm for different number of samples. The data sets in *x*‐axis of Figure 5 are arranged in ascending order of the number of samples present in each data set, that is, the number of samples in GSE17681, GSE17846, GSE28700, GSE24709, GSE21036, and GSE31408 data are 36, 41, 44, 71, 141, and 148, respectively.

From all the results reported in Figure 5, it is seen that different error rates such as *AE*, *B*1, and *B*.632+ do not depend on the number of samples present in the data set, rather, they depend on the distribution of the samples in different classes or categories. For example, although the number of samples in GSE17846 and GSE28700 data sets is almost equal, that is, 41 and 44, respectively, there is a significant difference in errors for these two data sets. The *B*.632+ errors for GSE17846 and GSE28700 data sets are 0.059 and 0.257, respectively. On the other hand, the *B*.632+ errors for GSE17846 data set with 41 samples and GSE31408 data set with 148 samples are 0.059 and 0.067, respectively.

**Figure 6:**
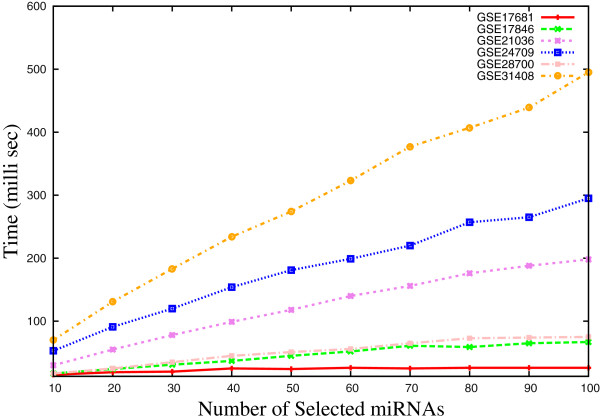
**Execution time of *μ*HEM algorithm on six data sets for different number of selected miRNAs.**

Figure 6 reports the execution time of the proposed *μ*HEM algorithm for different number of selected miRNAs. Results are presented for all six miRNA data sets by varying the number of selected miRNAs from 10 to 100. From all the results reported in Figure 6, it can be seen that the execution time of the proposed algorithm is directly proportional to the number of selected miRNAs, total number of miRNAs and samples.

### Importance of B.632+ error rate

**Figure 7:**
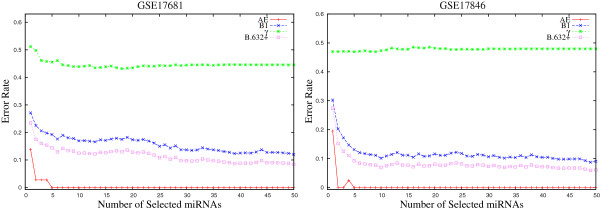
Different error rates of the proposed algorithm on GSE17681 and GSE17846 data sets obtained using the SVM averaged over 50 random splits.

**Figure 8:**
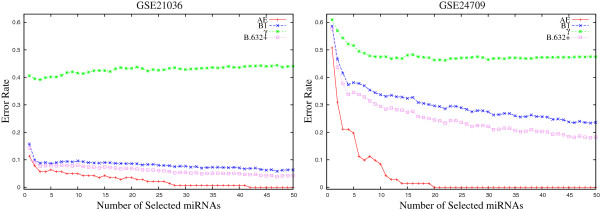
Different error rates of the proposed algorithm on GSE21036 and GSE24709 data sets obtained using the SVM averaged over 50 random splits.

This section establishes the importance of using *B*.632+ error rate over other types of errors such as apparent error (*AE*), no‐information error rate (*γ*), and bootstrap error (*B*1). Different types of errors on each miRNA expression data set are calculated using the SVM for the proposed method. All the results are presented for the optimum values of *ω* considering *d*=50. Figures 7 and 8 represent various types of errors obtained by the proposed algorithm on GSE17681, GSE17846, GSE21036, and GSE24709 data sets as examples. From Figures 7 and 8, it is seen that different types of errors decrease as the number of selected miRNAs increases. Similar results are also found for both GSE28700 and GSE31408 data sets. For all six data sets, the *AE* attains consistently lowest value, while *γ* has highest value. On the other hand, the *B*1 has smaller error rate than *γ* but it is higher than the *AE*. Moreover, the *B*.632+ estimate has smaller error rate than the *B*1 but higher than the *AE*.

Table 2 reports the minimum values of different errors, along with the number of miRNAs required to attain these values. From all the results reported in this table, it can be seen that the *B*.632+ estimator corrects the upward bias of *B*1 and downward bias of *AE*. Also, it puts more weight on *B*1 in situation where the amount of overfitting as measured by (*B*1−*AE*) is relatively large. It thus is applicable in the present context where the prediction rule generated by the SVM may be overfitted.

**Table 2:** **Comparative analysis of different types of errors for *μ*HEM algorithm**

**Microarray**	***AE***		***B1* Error**		***γ* Error**		***B.632+* Error**	
**data sets**	**Error**	**miRNAs**	**Error**	**miRNAs**	**Error**	**miRNAs**	**Error**	**miRNAs**
GSE17681	0.000	5	0.120	50	0.432	18	0.084	50
GSE17846	0.000	2	0.087	49	0.469	5	0.059	49
GSE21036	0.000	42	0.058	47	0.391	3	0.039	47
GSE24709	0.000	20	0.234	49	0.462	22	0.180	49
GSE28700	0.000	25	0.306	4	0.463	37	0.257	4
GSE31408	0.000	44	0.098	2	0.383	10	0.067	50

### Comparative performance analysis

**Figure 9:**
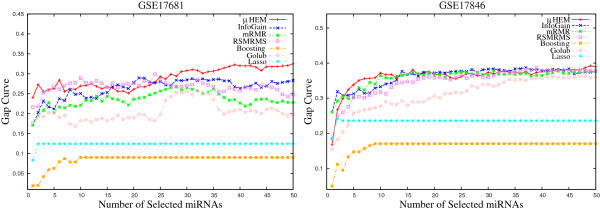
Gap curve obtained using different methods on GSE17681 and GSE17846 data sets averaged over 50 random splits.

**Figure 10:**
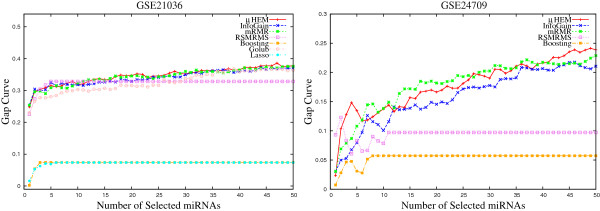
Gap curve obtained using different methods on GSE21036 and GSE24709 data sets averaged over 50 random splits.

**Figure 11:**
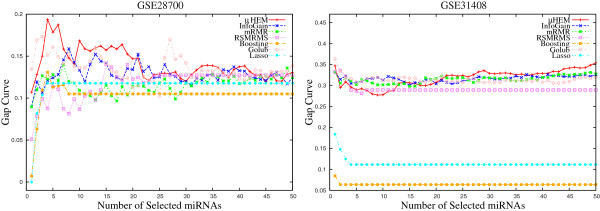
Gap curve obtained using different methods on GSE28700 and GSE31408 data sets averaged over 50 random splits.

**Figure 12:**
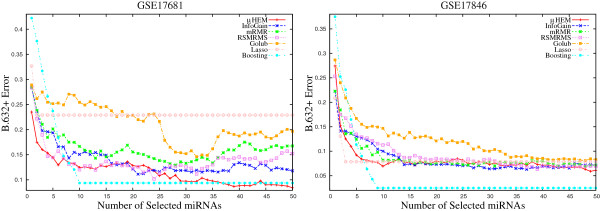
***B.632+* errors of the SVM obtained using different methods on GSE17681 and GSE17846 data sets averaged over 50 random splits.**

**Figure 13:**
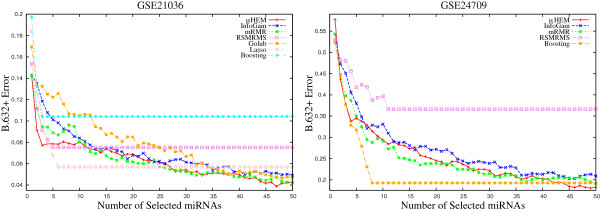
***B.632+* errors of the SVM obtained using different methods on GSE21036 and GSE24709 data sets averaged over 50 random splits.**

**Figure 14:**
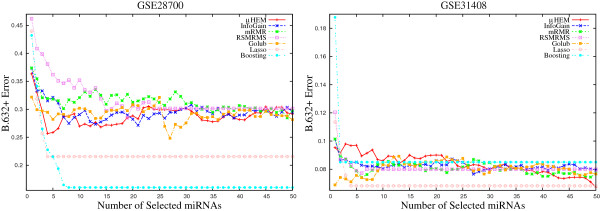
***B.632+* errors of the SVM obtained using different methods on GSE28700 and GSE31408 data sets averaged over 50 random splits.**

This section compares the performance of the proposed *μ*HEM algorithm with that of InfoGain [44], mRMR algorithm [45], method proposed by Golub et al. [46], RSMRMS algorithm [9], boosting [47], and lasso [48]. Table 3 and Figures 9, 10, 11, 12, 13, and 14 present different error rates obtained by various feature selection algorithms on six miRNA expression data sets.

**Table 3:** Comparative performance analysis of different algorithms

**Microarray**	**Algorithms**	**Apparent error**		***B1* Error**		**Gap estimate**		***B.632+* Error**	
**data sets**	**/Methods**	**Error**	**miRNAs**	**Error**	**miRNAs**	**Error**	**miRNAs**	**Error**	**miRNAs**
	Golub et al.	0.000	19	0.194	32	0.258	32	0.146	32
	Lasso	0.056	2	0.266	2	0.125	2	0.229	2
	Boosting	0.000	5	0.113	10	0.090	10	0.094	10
GSE17681	InfoGain	0.000	6	0.154	21	0.290	21	0.111	21
	mRMR	0.000	10	0.175	28	0.267	28	0.129	28
	RSMRMS	0.000	8	0.142	24	0.299	24	0.102	24
	*μ*HEM	0.000	5	0.120	50	0.325	50	0.084	50
	Golub et al.	0.000	6	0.116	48	0.363	48	0.081	48
	Lasso	0.024	3	0.102	3	0.241	2	0.079	3
	Boosting	0.000	4	0.037	9	0.170	9	0.025	9
GSE17846	InfoGain	0.000	7	0.093	37	0.387	37	0.063	37
	mRMR	0.000	3	0.101	48	0.379	48	0.069	48
	RSMRMS	0.000	2	0.093	39	0.386	39	0.064	39
	*μ*HEM	0.000	2	0.087	49	0.392	49	0.059	49
	Golub et al.	0.000	35	0.069	48	0.368	39	0.047	48
	Lasso	0.043	5	0.061	6	0.074	6	0.057	6
	Boosting	0.099	3	0.107	3	0.074	3	0.104	3
GSE21036	InfoGain	0.000	39	0.073	50	0.372	44	0.049	50
	mRMR	0.000	19	0.064	49	0.376	50	0.043	49
	RSMRMS	0.050	5	0.089	5	0.328	5	0.075	5
	*μ*HEM	0.000	42	0.058	47	0.386	47	0.039	47
	Boosting	0.099	8	0.211	8	0.057	8	0.192	8
	InfoGain	0.000	26	0.257	45	0.218	46	0.203	45
GSE24709	mRMR	0.000	24	0.245	50	0.229	50	0.191	50
	RSMRMS	0.141	11	0.402	11	0.123	2	0.366	11
	*μ*HEM	0.000	20	0.234	49	0.241	49	0.180	49
	Golub et al.	0.000	27	0.300	27	0.173	3	0.248	27
	Lasso	0.045	4	0.251	4	0.118	4	0.215	4
	Boosting	0.023	7	0.191	8	0.131	4	0.160	8
GSE28700	InfoGain	0.000	35	0.309	8	0.159	8	0.271	21
	mRMR	0.000	21	0.333	49	0.140	7	0.285	49
	RSMRMS	0.023	34	0.331	19	0.140	15	0.285	19
	*μ*HEM	0.000	25	0.306	4	0.194	4	0.257	4
	Golub et al.	0.000	36	0.073	1	0.364	1	0.069	1
	Lasso	0.061	3	0.072	4	0.184	1	0.068	4
	Boosting	0.081	2	0.087	2	0.085	1	0.085	2
GSE31408	InfoGain	0.007	20	0.090	9	0.331	1	0.077	27
	mRMR	0.000	37	0.094	6	0.331	1	0.074	6
	RSMRMS	0.061	2	0.086	6	0.336	2	0.077	6
	*μ*HEM	0.000	44	0.098	2	0.354	50	0.067	50

#### AE and B1 error

Table 3 compares the best performance of different feature selection algorithms based on the error rate of the SVM. From the results reported in Table 3, it is seen that the best *AE* for each miRNA data set is same for most of the algorithms. Both proposed *μ*HEM algorithm and mRMR method attain the best *AE* value for all data sets, while the method proposed by Golub et al. and InfoGain achieve it for five data sets and boosting and RSMRMS method attain this value on two data sets. However, the *μ*HEM achieves the best *AE* value with lower number of selected miRNAs than that obtained by other methods on GSE17681, GSE17846, and GSE24709 data sets, while mRMR method attains it for GSE21036 and GSE28700 data sets and the method proposed by Golub et al. on GSE31408 data set. On the other hand, the boosting method attains lowest *B*1 error rate in four cases out of total six data sets, while the *μ*HEM method and lasso achieve it only for GSE21036 and GSE31408 data sets, respectively.

#### Gap estimate

However, according to Efron and Tibshirani [37], the bootstrap approach (*B*1) overestimates the error. In this regard, the *Gap* function [49] is generally used to know whether the obtained *B*1 error is smaller than that would be expected by chance, if the distribution of the class‐membership label of the sample did not depend on its feature vector. The *Gap* function represents the difference between no‐information error (*γ*) and bootstrap error (*B*1), and is defined by

(29) Gap=γ−B1.

The larger value of *Gap* function indicates that the obtained or observed *B*1 error is significantly lower than that of expected by chance. Figures 9, 10, and 11 depict the gap curves, which highlight the difference between *γ* and *B*1 errors obtained using different algorithms on six miRNA data sets. From the results reported in these figures, it can be found that the *Gap* estimate increases with the increase in the number of selected miRNAs, irrespective of the algorithms and data sets used. Also, the *Gap* function always achieves significantly higher values for the proposed *μ*HEM algorithm, while for both boosting and lasso, the gap estimate is very low. Table 3 compares the best values of the *Gap* function obtained using different algorithms. All the results reported here confirm that the proposed algorithm attains highest values of *Gap* function in five cases, while the method proposed by Golub et al. achieves it only for GSE31408 data set.

#### B.632+ error

Finally, the performance of different algorithms is compared with respect to the *B*.632+ error. According to Efron and Tibshirani [37], the *B*.632+ error corrects the upward bias in bootstrap error with the downwardly biased apparent error. Figures 12, 13, and 14 report the variation of the *B*.632+ error for different number of selected miRNAs obtained by several feature selection algorithms on six miRNA expression data sets. From the results reported in Table 3 and Figures 12, 13, and 14, it can be seen that both boosting and lasso are useful to select a very small number of miRNAs, but not always appropriate to achieve lowest *B*.632+ error rate. The *μ*HEM algorithm attains lowest *B*.632+ error rate of the SVM classifier for GSE17681, GSE21036, GSE24709, and GSE31408 data sets, while boosting achieves it only on GSE17846 and GSE28700 data sets. The better performance of the proposed *μ*HEM method is achieved due to the fact that it provides an efficient way to compute degree of dependency of class labels on feature set in approximation spaces. In effect, a reduced set of relevant and significant miRNAs is being obtained using the proposed *μ*HEM method.

#### Execution time

**Figure 15:**
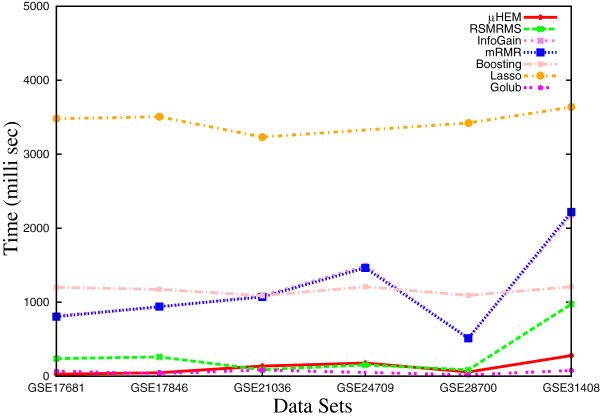
Execution time of different algorithms on six miRNA expression data sets.

Moreover, Figure 15 compares the execution time of different algorithms for six data sets. From the results reported in Figure 15, it can also be seen that the execution time of the proposed algorithm is significantly lower than that of most of the methods, irrespective of the data sets used. However, the execution time of the method proposed by Golub et al. is slightly lower than that of the proposed method. The lower execution time of the proposed algorithm is achieved due to its low computational complexity to compute the relevance and significance with respect to the number of selected miRNAs, total number of miRNAs and samples in microarray data set.

### Biological significance analysis

This section presents the biological significance of some miRNAs those are selected by the proposed *μ*HEM algorithm for GSE21036 data set as an example. The manually curated database, termed as miR2Disease [50], is used here to biologically validate the results obtained by the *μ*HEM algorithm. This database aims at providing a comprehensive resource of miRNA deregulation in various human diseases.

In GSE21036 data set, miRNA expression profiling has been done to understand the role of miRNAs that are responsible for the genesis and progression of prostate cancer [40]. The *μ*HEM algorithm selects a set of differentially expressed miRNAs from each bootstrap sample of GSE21036 data set. A set of nine miRNAs, consisting of **hsa‐miR‐145**, **hsa‐miR‐25**, **hsa‐miR‐153**, **hsa‐miR‐143**, **hsa‐miR‐19a**, **hsa‐miR‐96**, **hsa‐miR‐663**, **hsa‐miR‐20a**, and **hsa‐miR‐182**, is identified from all bootstrap samples of GSE21036 data set. Among them, four miRNAs, namely, **hsa‐miR‐19a**, **hsa‐miR‐20a**, **hsa‐miR‐663**, and **hsa‐miR‐182**, are identified by the *μ*HEM algorithm only, not by other feature selection algorithms.

One of the distinct characteristics of prostate cancer is over‐expression of the ERG proto‐oncogene. Several independent target prediction methods have indicated that the $3^{{}^{\prime }}$ untranslated region of the ERG mRNA is a potential target of **hsa‐miR‐145**. The **hsa‐miR‐145** is consistently down‐regulated in prostate cancer. In [51], it has been shown that the ERG $3^{{}^{\prime }}$ untranslated region is a regulative target of **hsa‐miR‐145** in vitro. From this observation it is suggested that the miRNA **hsa‐miR‐145** leads to progression of prostate cancer. The down regulation of **hsa‐miR‐145** is also mentioned in [52,53].

In [54], it has been shown that the **hsa‐miR‐20a** is over expressed in prostate cancer. Moreover, Sylvestre et al. described an over expression of **hsa‐miR‐20a** in the human prostate cancer cell line PC3 using PCR [55]. Volinia et al. recorded an up‐regulation of **hsa‐miR‐20a** in prostate cancer tissue using a microarray assay [56]. The identified function of **hsa‐miR‐20a** is the modulation of the translation of the E2F2 and E2F3 mRNAs via binding sites in their $3^{{}^{\prime }}$‐untranslated region [55], which supports the oncogenic behavior of **hsa‐miR‐20a**. The over expression of **hsa‐miR‐20a** reduces apoptosis in the prostate cancer cell line [55]. As suggested in [56] and miR2Disease, the **hsa‐miR‐25** is also up‐regulated in prostate cancer.

In [57,58], it is shown that **hsa‐miR‐143** expression is clearly down‐regulated during prostate cancer progression. ERK5 is known to promote cell growth and proliferation in response to growth factors and tyrosine kinase activation. Therefore, persistent decreased levels of **hsa‐miR‐143** in cancer cells may be directly involved in carcinogenesis through activation of the mitogen‐activated protein kinase (MAPK) cascade via ERK5. Taken together these findings suggest that **hsa‐miR‐143** could be a tumor suppressor and a potential novel diagnostic or prognostic marker in prostate cancer.

According to Hirata et al. [59], the **hsa‐miR‐182** regulates FOXF2, RECK and MTSS1 genes and is therefore over expressed in prostate cancer. They have also shown experimentally that these three genes are potential targets of the **hsa‐miR‐182** and play important role in progression of prostate cancer. Another miRNA, **hsa‐miR‐96**, is shown to be over expressed in prostate cancer as mentioned in [60].

## Conclusion

The contribution of the paper is two fold, namely, 

1. the development of the *μ*HEM algorithm for miRNA selection, integrating the merits of rough sets and hypercuboid equivalence partition matrix; and

2. demonstrating the effectiveness of the proposed algorithm, along with a comparison with other algorithms, on several real life miRNA expression data sets.

The concept of hypercuboid equivalence partition matrix is found to be successful in selecting relevant and significant miRNAs of real valued microarray data sets. This formulation is geared towards maximizing the utility of rough sets and hypercuboid approach with respect to insilico identification of differentially expressed miRNAs. The results obtained on six miRNA data sets demonstrate that the proposed method can bring a remarkable improvement on miRNA selection problem, and therefore, it can be a promising alternative to existing models for prediction of class labels of samples. All the results reported in this paper demonstrate the feasibility and effectiveness of the proposed method. The new method is capable of identifying effective miRNAs that may contribute to revealing underlying etiology of a disease, providing a useful tool for exploratory analysis of miRNA data.

## Availability and requirements

**Project name:***μ*HEM (Differentially expressed microRNA selection method)**Project home page:** www.isical.ac.in/ ∼bibl/results/mihem/mihem.html**Operating system:** developed on Linux (Ubuntu 12.04 LTS)**Programming language:** C

## Competing interests

The authors declare that they have no competing interests.

## Authors’ contributions

SP designed the current work. PM developed the concept of rough set based miRNA selection algorithm. SP implemented it and applied on different miRNA expression data sets. Both SP and PM analyzed the results and prepared the manuscript. Both authors read and approved the final manuscript.
